# A Treatise on the Nervous Diseases of Women; Comprising an Enquiry into the Nature, Causes, and Treatment of Spinal and Hysterical Affections

**Published:** 1841-07

**Authors:** 


### Art. IV.

*A Treatise on the Nervous Diseases of Women; comprising an Enquiry into the Nature, Causes, and Treatment of Spinal and Hysterical Affections.* By Thomas Laycock, m.d.—*London*, 1840. 8vo, pp. 356.

This volume being on a very important subject, we shall enter into a somewhat minute examination of its contents. These are very systematically arranged under the three general heads of Special Physiology, General Pathology and Therapeutics, and Special Pathology and Therapeutics, each part being subdivided into numerous chapters.

Part I. The first chapter of the first part is occupied with Principles and Definitions; and this is divided into two sections, of which the first includes what are considered by Dr. Laycock the general principles of the class of diseases of which he treats. These principles are four:

1. *The nervous system is the seat of hysteric diseases.* This is established, in the opinion of Dr. Laycock, by the "concurrent testimony" of all modern writers. Now, as we by no means assent to the general proposition that "what everybody says must be true," we are not prepared to surrender our own judgment to the overpowering force brought up in array by our author; and we think he shows a little indefiniteness in the ideas he attaches to the term "seat of disease." That the phenomena of hysteria are principally produced through the instrumentality of the nervous system cannot be questioned; but it does not follow that it is the part of the structure primarily affected. Indeed, Dr. Laycock himself defines the morbid state he is about to discuss as one in which there is no organic disease (by which we suppose that he means no structural change) in the nervous system. As we are not believers in the possibility of the occurrence of any morbid phenomena, without a disorder either of the structure exhibiting them or of the stimuli exciting them, we must ask Dr. Laycock whence the disordered action of the nervous system witnessed in hysteria originates. If in some primary change in the nervous system itself, then there *is* an organic disease; if from sympathetic relation with some other organ, then the nervous system is *not* the seat of the disease. We believe that from either of these sources, hysteria may develope itself. We are perfectly ready to go along with Dr. Laycock in the affirmation that the phenomena of hysteria immediately arise from a disordered condition of the nervous system; and this is all that he requires for his subsequent argument.

2. We are compelled to take exception also to his second principle, that *hysteria is peculiar to females;* for, as he himself admits, the phenomena of this disease occasionally present themselves in the male sex. According to Dr. Laycock, this exception serves but to prove the rule; but if the rule be understood to mean, as the words manifestly import, that there is something in the nervous system of women which renders them alone susceptible of the disease, it is obviously untenable.

3. The third principle, that *women of susceptible nervous system are more liable than others,* needs no comment, since universal observation supports it.

4. The last, however, that *hysteric diseases appear only during that period of life in which the reproductive organs perform their functions,* may require, like the second, a little qualification for exceptional cases. We trust that in these remarks we shall not be thought guilty of a carping and merely verbal criticism. Were these principles enunciated a little less *ex cathedrâ*, we should have felt ourselves less called upon to animadvert upon them; but the somewhat dogmatic style in which they are put forth challenges doubt as to their universality. A few statements of a similar character occur elsewhere, such as the following, with which we can by no means accord: "The advance in our acquaintance with the anatomy and functions of the brain and nerves, made during the last thirty years, is such as to render it certain that ultimately all generalizations, as well pathological as physiological, must be solely based upon the anatomy and physiology of the nervous system." Our opinion is just the contrary; for we hold that the tendency of physiological science at the present time is to reduce the vast amount of phenomena which, for want of some better explanation of them, have been regarded as originating in the nervous system, and to distinguish those which are *influenced by* that system from those which are *essentially dependent* upon it. We may refer, in illustration, to what we have formerly said upon the physiology of the capillary circulation, and the pathology of inflammation, (see vol. VIII., p. 188, &c.)

In the second section of the first chapter are discussed the General Relations of the Reproductive Organs; and here we find ourselves able to go along with our author with little exception. The reproductive organs are defined as essentially consisting of the ovaria in the one sex and the testes in the other, for which adequate reasons are given. The following very just observation concludes this section:

"It is by no means certain that all the sexual differences depend upon the sexual organs; on the contrary, it is highly probable that there is an organization and development of the primitive tissues, to which these are subservient; in short, that the ovaries and testicles being subject in common with the whole system to this primary impulse or nisus, owe to it their own distinctive characters, and have the secondary influences they exert modified in such a manner in consequence, as to bring out the special difference of the sexes, as seen in the presence, texture, and configuration of certain special organs." (p. 13.)

This view is far more conformable to the general analogies of nature than that which supposes the sex to be determined by an *accidental* enlargement or contraction of certain arterial branches during the progress of development.

In chap. ii. are displayed the *Special Relations of the Reproductive Organs*, which are very systematically treated of under the following heads: 1. Relations to the Cutaneous Products, in regard to the colours, appendages, subtegumentary fat, and odours, peculiar to each sex in different tribes of animals; all of which peculiarities are the most strongly marked at the period of greatest sexual excitement. The extent of the subject of course prevents anything more than a sketch from being here admissible; and this is very clearly given. 2. The Pathological Relations of the Cutaneous Products to the Ovaria and Testes. Under this head a great number of interesting facts are brought together relative to the changes which occur in the skin and its appendages, in consequence of disease, removal, or senile loss of function, of the reproductive organs. 3. The Physiological and Pathological Relations of the Ovaria and Testes to the Lumbar Region. "There is an influence," says Dr. Laycock, "exerted by the sexual organs on the muscular and osseous structures connected with this region, which is possessed by no other viscus; this is very distinctly shown by the difference of size of the male and female pelvis and abdomen." Now it appears to us that this doctrine is completely at variance with that which we just now quoted with approbation; since the larger size of the pelvis cannot be regarded as a *consequence* of the development of the ovaria, it being one of the concurrent changes originating in the same general nisus. 4. The Physiological and Pathological Relations of the Ovaria and Testes to the Thoracic Region. The general relation between the sexual organs and the neck and throat has been a matter of common observation from the time of Hippocrates downwards. A superior size of the neck and thoracic region is observable in the greater proportion of male animals; though there are some curious exceptions. The contained organs, too, are more developed; and the superior power of the voice of the male, almost throughout the animal kingdom, together with the approximations to the contrary character which occur in both sexes under the influence of sexual disease, attest in a remarkable manner the relation between them. Certain diseases of the thoracic viscera are much more frequent in men than in women; such are angina pectoris, aneurism of the heart and large vessels, and thoracic asthma. The relations of the glandular structures of this region to the sexual system are very remarkable. They are elucidated rather by pathological phenomena than by comparative observation of the lower animals. The affection of the testes in man and of the mammæ of women (perhaps also of the ovaria) as a consequence of parotitis, and the increased secretion from the salivary glands, are familiar instances. 5. The Physiological and Pathological Relations of the

Ovaria and Testes to the Organs immediately subservient to Reproduction. These, in order to avoid repetition, are only partially discussed here; the principal attention being given to the phenomena of menstruation, on which the most recent information is given. Dr. Laycock reverts to the doctrine of the older authors, in considering the period of menstruation as analogous to that of heat in the lower animals; there being at this period an excited state of the ovarium, which is accompanied by a shedding of ova ready to be fructified. "But we shall find," he adds, "that this periodic movement is not limited to the ovaria; but that it is an affection of the general system in which the ovaria partake; and that it is through these the secondary system in connexion with them is influenced, and all the attendant phenomena (those of menstruation) excited." (p. 43.)

In chapter iii. *the Periodic Movements in the Reproductive Organs of Woman* are considered. The most obvious of these, that of menstruation, is treated of in section i. This is considered by Dr. Laycock as only an example of a large class of changes in the human system, which are governed by certain *periodic* influences.

"Not only are there periodic influences ever acting upon man which are derived from without, but from the moment of his conception (and perhaps before,) series of changes commence, enchained by definite laws, and depending for their due development upon agencies seated in the individual organism. The more prominent links of these series are too obvious to escape notice; and the septennial phases of man's existence were remarked at an early period. The quotidian movement, as indicated by sleep and wakefulness, and the regular daily recurrence of appetites and functions, could not fail to be observed; and the monthly movement, from its marked occurrence in females, was as easily distinguishable. Since the phenomena of these periodic movements are open to observation, there can be no doubt that it is possible to arrive at a knowledge of the principles by which they are governed." (p. 45.)

In pursuit of this enquiry, our author brings together a number of instances in which menstruation occurs at regular periods differing from that ordinarily known, as, for instance, two, three, or five weeks; and he concludes that "although there be no flow of the catamenia every week, yet a change takes place at that period in many females, more or less felt as the system is more or less robust; but strongly shown when excitability of the nervous system, either general or local, exists, and the phenomena of disease are developed, as paralysis, spasm, insanity, or hemorrhages." We should like to know a little more minutely the evidence upon which this rather novel conclusion is founded. If true, it is certainly important; but we are by no means satisfied of its validity. That the menstrual change occurs during pregnancy was maintained by the ancients; and Dr. Laycock thinks it extraordinary that the doctrine should have fallen into neglect. There is a looseness of statement, however, on this point, which leaves us in doubt as to our author's meaning. Does the menstrual change in the ovaria take place as well during pregnancy as in the previous condition? Or is it only the attendant phenomena, the pain in the loins, the tumescence of the mammæ, &c. which occur? Dr. Laycock endeavours to strengthen his position as to the *weekly* excitement of the sexual system of the human female by a reference to the periods of utero-gestation of the lower animals, a large and valuable collection of information on which head, as also on the period of incubation in oviparous animals (which is evidently an analogous state), is contained in section ii. of this chapter. In spite of several exceptional cases, Dr. Laycock concludes that in all mammalia the period of utero-gestation may be stated in terms of *weeks,* in other words, is a definite multiple of the hebdomadal period; and that the period of incubation in birds is normally the same, although liable to be influenced by temperature, domestication, and other circumstances.

In the third section are collected facts relating to the Periodic Changes of Insects, which are regarded by Dr. Laycock as strengthening his position, that periodic changes occur in animals at periods of weeks or multiples of weeks. These facts are not stated in sufficient number or with sufficient definiteness to be satisfactory to us; but, as Dr. Laycock remarks, a *rigidly exact* series of observations on the time occupied between the various changes and developments of insects is a desideratum.

In the fourth section is briefly noticed the Periodic Recurrence of the "Heat" of Mammals, already spoken of as analogous to the menstruation of the human female. This also is known to recur at definite intervals; and although usually absent during pregnancy, yet it occasionally (like the catamenia in some women) displays itself throughout. The *rutting* of the males is somewhat analogous to the heat of the female; and its periodical recurrence appears to be as regular. In both, according to Dr. Laycock, the hebdomadal period or its multiples may be observed.

In the fifth section are considered the Vernal and Autumnal Changes which all living beings undergo, and which seem especially to affect the sexual system. The following is Dr. Laycock's general summary of the questions discussed in this chapter:

"First, that at hebdomadal periods or their multiples, a change occurs in the system of all animals; and that at puberty, and each menstrual period subsequently, the sexual organs of the female share in this change in common with the whole system, the phenomena resulting [in the human and some other species] being those of menstruation; and that, consequently, the flow of the catamenia is no more than a local effect of the general cause. Second, that the system of all animals undergoes changes about the vernal and autumnal equinoxes, and probably about the summer solstice, in which the generative organs and their dependencies partake; and that the causes of these changes modify in mankind all diseases, but those more particularly connected with the reproductive functions." (p. 70.)

A short chapter (iv.) next follows on *the Relations of the Reproductive Organs to the Nervous System in general;* a subject which is obviously of very extended scope, and which might, we think, have been with advantage treated a little more fully by our author. Among the influences on the system at large produced by the state of the reproductive functions, Dr. Laycock notices the pugnacious propensities remarkable in the males, and the artfulness of the females, qualities which are not without their parallel in the human race. Another remarkable effect of the change in the system at large, induced during the performance of the reproductive functions, is a loss of appetite or cessation of its indulgence, such as is peculiarly manifested in insects. The influence of physical love on the appetite of men and women is a matter of daily observation; and bulimia, pica, and strange longings are morbid modifications of the appetite, and belong to the same class of phenomena as this anorexia, —like it, being characteristic of the pregnant, chlorotic, and hysterical female. The whole nervous system is excited by the sexual stimulus, as much as by medicines which have a direct and powerful influence on it. It has been remarked that in frogs, just before the period of copulation in spring, and in the autumn also, the nervous system is endowed with a peculiar irritability; so that the slightest touch will at these times produce the effects of powerful excitants at others. A corresponding state occurs in the morbid sexual excitement of women, known as nymphomania; in which the system becomes endowed with all the irritability observed in hydrophobia, the slightest touch (as in frogs) inducing tetanic spasms. In satyriasis the symptoms are somewhat analogous. Our practical readers will doubtless call to mind phenomena of a similar description in milder cases of hysteria; and it is interesting to be aware that they find a parallel in the natural condition of another species.

In chapter v. are discussed the *Mental and Corporeal Peculiarities of Woman.* The *affectability* of her nervous system is first alluded to, as the governing principle, so to speak, of her constitution; an observation in the truth of which observers of human nature in all ages have concurred. This peculiarity is at the same time the cause of her weakness and frailty, and of the strength and nobleness of her character under particular circumstances. Dr. Laycock then adverts to those peculiarities of her configuration, which, though influenced perhaps by the development of the ovaria, are by no means dependent on them; and quotes Meckel and others in regard to the very early period of fœtal development at which these distinctive marks may be perceived, a period anterior to that at which any perceptible difference in the sexual organs manifests itself. Even at a more advanced period, there is still a manifest difference in the corporeal structure of the sexes, long before the period at which the generative system begins to exert its peculiar influence; this has been well shown by the researches of Quetelet. Further, the male and female which are deprived of these organs at a very early period retain these peculiarities through the whole of life; thus the castrated boar is readily known from the spayed sow by his larger limbs and more massive bones, although both may have belonged to the same litter, and may have been mutilated at the same early age. From these facts, Dr. Laycock is inclined to the opinion of Cabanis, that the peculiarities of the sexes, whether mental or corporeal, including those referred to the genital organs, depend upon some particular organization of the primitive nervous system. Now this hypothesis assumes that the development of the nervous system governs that of all other parts, a doctrine which is neither conformable to analogy nor to observed facts. If, for example, we extend our survey to the sexual system of plants, and investigate the conditions under which the sexes of the diœcious species are respectively produced, we find them subject to no influence from a nervous system; and there have been instances of abnormal development of human fœtuses in which the absence of both brain and spinal cord have demonstrated the essential independence, on the part of the formative processes, of the agency of the nervous system. We have stopped to advert again to this question of the influence of the nervous system, because we deem it of the first importance that, if principles are to be sought out and followed at all, they should be correct ones. Dr. Laycock does not rest satisfied, however, with this doctrine; but, perceiving that it offers nothing very tangible for consideration, he directs attention to the vascular system as the one which presents the most evident differences. We think that in this course he is more theoretically correct than he himself supposes; for on the functions of the vascular system depend alike the first formation of nervous matter and the development of its peculiar properties, and also the excitement of these properties to action; and it is here, therefore, that we should look for the first cause of the peculiarities of that action. In the following remarks there is much justice and acuteness:

"It is clear from these statements that the vascular, no less than the nervous, system is implicated in hysterical disorders; and it is equally obvious that the term hysteria is singularly inapplicable, and the term nervous diseases altogether vague. Perhaps a good general term for the whole class of hysteric and hypochondriacal diseases would be *neuræmia*, (*νεῦρον* and *αἷμα*), and *neuræmic*; being distinguished from organic diseases of the nervous system in this, that in the latter it is not so much the blood as the blood-vessels which are diseased, as in paralysis, apoplexy, &c.; thus *neuræmic* neuralgia would be distinguished from real tic-douloureux dependent on organic disease. It is true one class passes insensibly into the other; but this happens in all classifications of vital structures and functions." (p. 84.)

The distinction here drawn is an important one. It has been repeatedly shown that the quality of the blood is lowered by frequent hemorrhage, and that this very condition predisposes to further hemorrhage, in consequence of the altered state of the tissues surrounding the vessels, the more ready permeation of the diluted fluid, and the tendency to stagnation which results from its diminished power of ministering to the proper functions of the capillary circulation, and of moving through these minute tubes. This condition of the blood is, therefore, a most fertile source of morbid action; and is nowhere more so than in the nervous system, the dependence of whose functions upon those of the vascular is so extremely close. Yet its results are often spoken of as *functional* diseases; as if there were any morbid action which had not *some* material cause.

The succeeding chapter, in which are considered some points in the metaphysics and physiology of the nervous system, is to our minds the least satisfactory of the whole work. There is a good deal of vague and pointless allusion to the doctrines taught in the ancient schools of philosophy, mixed up with speculations respecting the molecular actions of matter, which do not seem to lead to any definite end; in fact we are at a loss to know what our author himself had in view.

In chap. vii. is given a general sketch of the present state of our knowledge of "the brain and sensitive nerves as the organs of consciousness;" and the next chapter contains, under the title of "the movements of animals in relation to consciousness," an outline of Dr. M. Hall's and Mr. Grainger's researches on the phenomena of reflex action. These topics are rather beyond the proper limits of such a work as Dr. Laycock's; but he has introduced them as preliminary to the subject of the next chapters,—"The instinctive actions in relation to consciousness: the brain subject to the laws of reflex action;"—and "the action of the will, and of internal and external stimuli, on the hemispherical ganglia." In the former of these it is shown that the actions commonly regarded as *instinctive*, and those resulting from the *direct* influence of the passions and emotions, are of the same general character as those termed reflex by Dr. M. Hall; being performed without the intervention of the will, through a system of nerves distinct from those which convey the influence of volition, and being immediately excited by external stimuli. On this question we have recently dwelt at length, and shall not therefore here revert to it.

Our author's chief object in the succeeding chapter is to direct attention to a class of phenomena, which he believes to constitute an important part of those ordinarily known as the nervous diseases of women, and which result from the power of the will, not only over the motor but over the sensorial fibres of the brain. This is exerted in the ordinary acts of memory, in which a change is voluntarily produced in the sensorial fibres, corresponding to that which the object itself, when presented to the organs of sense, had occasioned. On this principle, Dr. Laycock considers, may be explained many of the phenomena of Mesmerism, which are at first produced by an act of the "sensorial will," but which subsequently become so easily excited, as to recur almost involuntarily, or upon some slight external stimulus. Dr. Holland has shown the importance of the same general principle, in the aggravating influence of attention to the state of the digestive system, on the part of hypochondriacal patients; and Dr. Laycock refers to the recent work of Professor Dubois upon hypochondriasis, as developing it still more fully. This writer divides the phenomena into three stages: in the first the mind only is affected; the patient is harassed by imaginary diseases, and concentrates his attention upon one or other of the viscera—hereby changes in their innervation are excited, when the second stage is developed; and in the third, these merely nervous affections terminate in organic disease of the affected organs. "The effect of the phenomena of Mesmerism," it is very justly observed by Dr. Laycock, "will be useful in this, that the attention of philosophers will now be more than ever directed to the action of the will on the sensorial fibres of the brain, and through these on the sensitive nerves,—to the laws by which it is governed, and to the changes in distant organs which follow its agency."

Our author next proceeds to consider "the pathological and physiological relations of the nervous system to the ovaria," to which chap. xi. is devoted; and he begins with a general survey of the pathological relations of the spinal cord, especially as regards those complex and puzzling phenomena which result from an irritation in some part of the body, very remote from that in which they manifest themselves. It has been long since remarked, that a disorder in any of the viscera was likely to affect the portion of the spinal cord with which it was connected, and thence to produce sympathetic feelings or movements in various parts of the body. Thus, intestinal or visceral irritation is well known to cause convulsions; and paraplegia has been seen to exist in several instances without appreciable disease in the spinal cord or its membranes; the kidneys being in a state of suppuration, and having produced a functional disturbance of the nervous centres with which they are connected. Not only may the system at large, or various parts of it, be thus morbidly affected by a local disorder, but its condition will be also very much influenced by the state of the mind, especially of the feelings and passions; which probably act upon the roots of the sensory nerves, and produce changes in them which are referred by the mind to their peripheral extremities, and which may (on the principle just referred to) become an actual cause of disease in the parts to which they are distributed. Dr. Laycock alludes to the singular march of erratic erysipelas, (well described by Dr. Graves) as easily understood on the supposition that the disease is caused by a morbid state of the nervous centres from which the nerves of the part take their origin; the disease advancing symmetrically, and invariably attacking in succession those parts of the skin which are supplied from the respiratory tract. If these general views be correct, it is very easy to understand how the connexion of the uterus and ovaria with the lumbar portion of the spinal cord should give rise to so great a variety of both functional and structural disorders; and how also these organs should be sympathetically affected by remote causes, operating through the passions and feelings of the mind, as well as more directly through the body. The value of the speculation, however, can only be determined by an extensive series of observations made with special reference to it.

Part II. *On General Pathology and Therapeutics.* In order to avoid repetition, as well as the appearance of ungraciousness towards our author in the too frequent dissent from his opinions, we shall, in commencing our notice of this portion of his work, state our general objections to some doctrines and some peculiarities of reasoning by which it is pervaded. When speaking of the periodical movements of the living body, we had to call in question the accuracy of Dr. Laycock's notion of hebdomadal terms. The evidence adduced in its favour is to our mind extremely feeble; whereas, nothing short of demonstration could sanction the adoption of a principle which would change the whole aspect of theory with respect to the diseases of women, and thence with respect to all disease, inasmuch as it would tend to subvert those general pathological views which have hitherto been considered as equally applicable to both sexes. For example, our author tells us that "the fourth day, and the seventh, eleventh, and fourteenth, are critical days, and connect the doctrine of crises with the menstrual period." (p. 150.)

Now, to say nothing of the arbitrary solution of a much disputed question—whether critical days have any real existence—we have here an hypothetical relation set up between certain crises, which, if they exist at all, are common to the diseases of both sexes, and a periodic function which is peculiar to one sex. If critical days be connected with menstruation in woman, what are they connected with in man? And if Dr. Laycock's view of the subject be just, must we not adopt perfectly distinct principles of pathology and treatment in fever as occurring in the two sexes?

Another remark we have to make is that our author appears to have adopted too implicitly, and applied too extensively, an idea of Dr. Henry Holland's, which we admit, nevertheless, to be well worthy of attention; namely, that many of the anomalous diseases of women may, in truth, be referrible to the gouty diathesis, which, being prevented by some peculiarity of the female constitution from developing itself in any of its more familiar forms, assumes others less readily cognizable.

The following sweeping assertion will sufficiently show the extent to which Dr. Laycock has been carried away by this notion:

"It may be stated here generally that, of the many cases related by authors as anomalous hysteric diseases, by far the greater portion were connected with a gouty diathesis, as indicated by the formation of calculi, by the occurrence of regular paroxysms of gout, and by the descent of the individual from gouty ancestors; they are cases, in fact, which would have been better understood and better treated, if they had been termed anomalous gout; but as the subjects were young females, they were, of course, set down as anomalous hysteria." (p. 163.)

Nothing can be more unsatisfactory than a *general* appeal to authors, when a new position is to be established or a disputed point settled.

Such premature pathological conclusions as these may naturally be expected to lead to questionable views of practice. Speaking of the material cause of gout, which he conceives to be an excess of urea or other constituent of the urine, Dr. Laycock remarks that, "When we consider the composition of this *materies morbi* in its general relations, many useful inferences, both theoretical and practical, may be deduced. Nitrogen is in the largest proportion, and the most important part of any of its constituents, and hence the utility of such muscular exercise in gout and hysteria as will 'use up' (if I may be permitted the phrase) the superabundant nitrogen present in the system. Hence also the propriety of a diminished consumption of animal food, and regular exercise as a prophylactic in both diseases; and hence also the applicability of the same medicinal agents to the cure of both." (p. 170.)

We suspect that the benefits arising from muscular exercise in the cases alluded to are attributable quite as much to the free distribution of the blood and the due appropriation of nervous power as to the consumption of nitrogen. Dr. Laycock's proposition that a diminished consumption of animal food is prophylactic of hysteria is too general to be either reasonably maintained or easily confuted, but we are quite sure that there is a very large proportion of cases to which it will not apply. We are equally sure that nothing but an hypothesis could have driven our author to so singular an assertion as that the same medicinal agents are applicable to the treatment of hysteria and of gout. The only legitimate use at present to be made of the presumed origin of some anomalous diseases of women in the gouty diathesis is to observe on a large scale, and to record numerically, the diseases prevalent among the females of gouty families. This might lead to the discovery of important relations; but we apprehend much evil from hasty assumptions on the subject. The speciousness and apparent extensibility of the notion in question are likely enough to render it popular, and we should not be much surprised, some time hence, to meet at every turn a clandestine gout skulking in neurotic garments, and to find colchicum the fashionable medicine of the day.

A third general objection we have to make to our author's manner of handling his subject is his proclivity to vague analogies. For example, he occupies a considerable space, and gives himself no little trouble, in showing a sort of affinity between the symptoms of hysteria and those produced by certain animal, vegetable, and mineral poisons; those caused by excessive loss of blood; and those attendant on chorea, tarantisma, laryngismus stridulus, and other convulsive affections; but what is this, after all, but making a long-winded announcement of the fact that the effects of morbid or deleterious causes which powerfully influence the nervous centres, have more or less resemblance to one another, inasmuch as they implicate the same organs and disturb the same functions? Analogies of a similar kind, might, with as much truth and as little utility, be shown to exist among various phlegmasiæ, local injuries, and other causes which severely disturb the functions of the vascular system.

The remarks we have already made may have prepared the reader for the last general exception we have to take against Dr. Laycock's mode of philosophising.

It may perhaps be deemed a Milesian piece of criticism on our part to make a *general* objection to *generalities;* we do so, however, to supersede the necessity of noticing all the instances, which we regret to say are not few, where five minutes' reflection would have preserved our author from making general assertions which he would find it extremely difficult to support by facts. Some passages, on which we shall have occasion to comment in the sequel, will be found amply to justify our present remarks.

Having made these preliminary strictures, we are in a condition to follow our author through the principal details of his subject. In chap. i. of part ii. he makes some general remarks on the nervous diseases of women, and expresses his conviction that "the best mode of threading the labyrinth in which these diseases are involved is, to avoid the usual scholastic method of treating them, and instead of considering their proximate, remote, predisposing, or exciting causes, their various modifications, and their general pathology, to examine into their physiological and pathological relations, taking as our guide the general principles of the division devoted to their special physiology." He proceeds in chap. ii. to treat of the "general relations of the nervous diseases of women to growth, development, and decline," and the first section is on the "relations to growth and development antecedent to puberty." We cannot say that we here perceive much direction in his remarks. He contends, perhaps justly, that the excitability of the cerebral hemispheres is less in infancy and childhood than in adult age, and supports his opinion by the remark of Sir B. Brodie, that the proportion of recoveries from wounds of the brain is especially small in adult patients. Dr. Laycock adds a table, giving the results of eighty-one cases of injury of the brain, collected from various publications, and from his own notes. It is as follows:

| | Whole number. | Recovered. | Died. |
|---|---|---|---|
| Patients under 10 years of age | 8 | 6 | 2 |
| Patients between 10 and 15 (inclusive) | 24 | 18 | 6 |
| Patients between 16 and 21 (inclusive) | 8 | 7 | 1 |
| Adults of all ages above 21 | 41 | 14 | 27 |
| Total | 81 | 45 | 36 |

All tables of this kind are valuable, but we do not think the one before us greatly favours the notion in support of which it is adduced. In the first place, we cannot admit the result of injuries of the brain as a fair measure of its excitability at different ages, because other organs as well as the brain undergo modifications by age, and the relation of the brain to these organs is also modified; and such modifications have probably great influence on the result of injuries of the brain as regards life or death, independently of the amount of damage sustained by the brain itself. Secondly, though the table shows that the mortality is greatest in adults, it does not make it least in infants; which it should do in order to bear out the views which we shall immediately find our author propounding. He regards dentition as a period of general development, and maintains that its painful or irregular progress may be much oftener an effect coexistent with convulsions, than a cause of the latter. Here we believe that he is correct; but when he endeavours to trace an analogy between the phenomena of the first dentition and those of hysteria, and declares that there is a peculiar state of the nervous system of infants during dentition, analogous to that at the commencement of menstruation, and its periodic return, we have only to say that we think some of our preliminary remarks here become particularly applicable. At the period of the second dentition, Dr. Laycock conceives that the brain has acquired a higher excitability, while the sensibility of the mucous membrane has diminished, and this in proportion to the growth of the individual: so that few of the phenomena which attend the first dentition are observed in the second: the exceptions to this rule being found in delicate, excitable children, whose development is retarded. These circumstances, combined with the influence of incipient puberty, occasion, according to our author, a diversity in the phenomena of nervous disease in the two sexes, convulsive affections assuming the form of epilepsy in boys and of chorea in girls. The latter affection he regards as the precursor of hysteria; in fact, as the hysteria of the female child: and he observes, that "it will be found accompanied by the same phenomena, *mutatis mutandis*, as accompany the first evolution of the teeth." (p. 136.)

The necessity for the qualification here introduced cannot be better shown than by recurring to the statement made by our author, only in the preceding page, that "few of the symptoms which accompany the first dentition are observed in the second." Now, since the phenomena of chorea are associated with those of the *second* dentition, and this presents phenomena widely different from the *first*, it appears to us that *mutatis mutandis* here amounts pretty nearly to *mutatis omnibus*, with which qualification we freely admit the analogy between chorea and the first dentition. Such contradictions as these are the inevitable consequences of reasoning, or speculating rather, on intricate and extensive subjects without sufficient data. We must further observe that Dr. Laycock's affirmation with respect to epilepsy, as the convulsive affection of boys and chorea of girls, is a very gratuitous one, and opposed to the best authorities, as well as to daily observation. With respect to the relative frequency of epilepsy in the two sexes, the statements of authors vary so much as to lead to the inference that the occurrence of the disease is not much influenced by sex, though it is probable, on the whole, that males are most subject to it.* On the other hand, though chorea

* Dr. Copland says, "The influence of *sex* is not remarkable; and is not manifested until after the second dentition. According to Esquirol and Foville, females are more subject to the disease after this epoch than males. At the end of 1813, 162 male epileptics were in the Bicêtre, and 289 female cases in the Salpêtrière. J. Frank found that of seventy-five patients, forty were females; but he agrees with Celsus, Heberden, is decidedly more frequent in females, the preponderance is not such as to justify us in regarding it as at all peculiar to them. Indeed Dr. Laycock himself supplies us with abundant proof to the contrary in a table constructed by M. Rufz, of the Hôpital des Enfans Malades. Out of 189 cases, fifty-one were males; and our author, after citing Drs. Manson, Heberden, and Elliotson, acquiesces after all in the common estimate, according to which the proportion of girls affected with chorea to that of boys is about three to one. It is unaccountable that a man of Dr. Laycock's sense should thus make round assertions, as if for the mere pleasure of disproving them himself. This would be of less consequence if the disproved assertions were excluded from the author's reasoning, but they too often continue to form an essential part of his argument.

We should mention, that the table by M. Rufz, published in the Archives Générales for February, 1834, is referred to by Dr. Laycock, to show that the liability to chorea increases as the period of puberty approaches. (pp. 135-6.)

Sect. ii. treats of the "relations to the period between puberty and marriage, or between the ages of fourteen and twenty-one." The author makes some observations on chlorosis as one of the earliest diseases of the period which commences with the evolution of the sexual organs, and then on the hysterical disposition which accompanies a more advanced state of development. He regards hysteria as capable of hereditary transmission, in which we agree with him; and he makes some good remarks on the influence of the present system of moral and physical education in deranging the nervous functions of young females. He notices the effect of inordinate impressions on the external senses, and especially of music when too ardently cultivated, and animadverts with justice on the general want of attention to these points.

The third and concluding section of this chapter contains some brief remarks, which need not detain us, on the "relations to the period between the age of twenty-one and the cessation of the catamenia."

Chap. iii., comprising only half a page, is on the "relations of the nervous diseases of women to the uterus and ovaria." The author very properly concludes that hysteria is by no means necessarily dependent on disease of the uterine system, and that chronic hysteritis is in many cases an effect, not a cause, of the hysterical state. He affirms also that when the reproductive organs are the seat of the exciting cause of hysteria, the morbid changes they exhibit resemble those induced by conception rather than by organic disease. He states that the most constant appearance observed in the bodies of those who have died of hysteria has been enlargement of the ovaria, with vesicles containing an albuminous fluid, or resembling the Graafian, that such were the appearances observed by Villermay in a young female who died of hysteria induced by fright, and that the same author cites Riolan, Blanchard, Binninger, Vesalius, Diemerbroeck, and Morgagni, as having witnessed somewhat similar changes. The evidence, we think, is here too slight to justify any and Soemmering, in believing that if a strict diagnosis were established between this and other convulsive diseases to which females are very liable, particularly several of those seizures described in the article *Convulsions,* the predominance would be found on the side of the males; and Drs. Cooke, Elliotson, and Cheyne, are of the same opinion." —*Dictionary of Prac. Med.*

general conclusion; we are not, however, inclined to dispute that of our author, being ourselves in a state of acknowledged nescience.

Chap. iv. is on "the relations of nervous diseases to periodic movements." It contains two sections: the first treating of the "relations to periodic movements dependent on cosmological influences;" the second of "relations to the weekly periodic movement." The author's observations on these subjects are rather indefinite, as might indeed be expected from the absence of all sufficient materials for reasoning; nevertheless, they may be read with advantage, as pointing out the recurrence of various forms of disease at various periodic terms, a series of phenomena which, for a long time to come, we should content ourselves with observing, recording, and comparing; any attempt at theory being in the present state of our knowledge entirely futile.

Chap. v. is on the "relations of nervous diseases to alterations in the composition of the blood." The author states that he does not here propose "to review all the extensive relations which nervous diseases bear to the composition of this fluid, but only to notice those points which are practically useful, or may lead to more enlarged views, or more correct observations." These points he conceives to be "the effects of blood-letting, and other exhausting agencies, of certain poisons which act by entering the circulation, and the phenomena of gout considered as being caused by a materies morbi circulating with the blood." (p. 152.) This chapter contains allusions to some curious cases, and the enumeration of a great variety of nervous phenomena produced by divers causes, and involved by the author in those analogies to which we have already represented him as, in our opinion, unduly addicted. Indeed our general remark on his proneness to vague analogy was more immediately suggested by the perusal of this chapter. Towards the conclusion of it, he dwells at considerable length on the relations of gout and hysteria, and their joint connexion with derangement of the renal functions. Having glanced, in a cursory manner, at a variety of instances illustrative of the gouty origin of certain nervous affections of men as well as women, he adverts particularly to the occasional occurrence of an erratic secretion of urine, or some of its constituents, both in gout and hysteria, referring to another part of his work for examples of such phenomena in the latter disease. We give the following rather long quotation, as affording a fair example of our author's style, and as conveying his ideas more distinctly than could be done by an abstract:

"The connexion between total suppression of the urine and gout, but especially between gout and gravel, has been frequently noticed. It is well known that all articles of diet which, either from their peculiar composition, or their action on the organs of assimilation, increase the quantity of lithic acid, will excite a fit of gout or gravel. The favorable termination of a fit of the gout is always accompanied by an increased excretion of lithic acid from the kidneys. Colchicum also, which is almost a specific for gouty diseases, increases this excretion; the quantity being nearly doubled, according to Professor Chelius, after the medicine has been taken for twelve days. The urine of gouty subjects is in general acid, but during the paroxysm it becomes alkaline or neutral, and so continues until the termination of the paroxysm, when it becomes loaded with the lithic acid deposit. Cases of gravel indeed are simply those in which the acid crystallizes so soon as excreted by the kidneys; in cases of gout the excretion is suspended; and these lithic formations, according to Dr. Prout, seldom occur before the age of forty, a period of life corresponding to that in which gout is first developed in those with the hereditary predisposition. Facts being such, the direct inference is, that the presence of an excess of lithic acid, or other of the urinary constituents, is the immediate cause of a paroxysm of gout; and that those individuals are of a gouty constitution who, from hereditary conformation of the organs of assimilation or excretion, either have an unusual quantity of uric acid produced, or a deficiency of power in the kidneys to eliminate it from the system, as maintained by Dr. Holland. And such inference is quite in accordance with all toxicological, and (less directly) with many pathological phenomena, apparently unconnected with gout. . . . . It is not generally known that Jahn found crystals of the gouty salts in the blood of gouty patients; a fact, however, of the greatest importance. Turck, a recent French author, attributes gout to the presence either of too much acid or too much alkali, which latter he connects with a set of acid and alkali secreting organs, and these with positive and negative electricity. While the preceding statements with regard to the pathology of gout illustrate and confirm the views taken respecting the ovarian origin of hysteric diseases, they open out a large field of enquiry into the relations existing between the ovaria and testes on the one hand, and the kidneys on the other, as well as between both and the two large classes of diseases I have just compared. According to the researches of Wolff and Rathke (confirmed by Muller), the formation of the kidneys in the embryo is preceded by a substance termed by Rathke Wolffian bodies, after their discoverer. Like the true kidneys in fishes and batrachian animals (to which they are analogous in structure) the Wolffian bodies extend along the whole length of the spine, from the heart to the end of the intestine; a fact, as we shall see from the next statement, highly interesting, because it proves the extensive connexion the genito-urinary system has with the spinal cord. In mammalia a vessel proceeds from these bodies, which opens into the vas deferens, or fallopian tube. In the higher vertebrata these false kidneys are used to contribute to the development of both the true kidneys and the testes and ovaria, the latter first appearing attached to their anterior margin by a fold of peritoneum. In the human embryo, at the seventh week, they are slender elongated bodies, situate below the kidneys, their permanent position in the lower vertebrata." (pp. 166-8.)

The above embryological sketch affords, as Dr. Laycock thinks, a key to the explanation of many anomalous symptoms and phenomena of gout, hysteria, and some diseases connected with them. "We can," he says "have no difficulty in conceiving how the kidneys may, in many instances, have distinct sympathies, similar to those of the ovaria and testes; or in understanding how the latter may modify the functions of the former, and so contribute to the establishment of diseases which originate in a defective elimination of the urinary constituents." (p. 168.)

We have intimated our conviction that Dr. Laycock's mind is too much possessed with the relation between gouty and nervous disease; nevertheless, regarding the question as an important one, we strongly recommend his observations to attentive perusal, since they display both ability and an extensive knowledge of his subject.

Chap. vi. treats of the "relations of the emotions, passions, and propensities." The first section is on the emotions and passions, the second on the propensities, and the third on the causes which modify the influence of the passions on woman. Dr. Laycock is not diffuse on any of these heads. Their full consideration would indeed occupy a voluminous work, and would require an immense range of observation and great analytical power on the part of the writer. Some of our author's remarks on imitative movements, in the second section, appear to us to merit attention, especially that relating to their excito-motory character; this subject, however, we must waive, for reasons stated in a preceding part of the present article.

In the same section we find one of those outrageous affirmations which we have encountered so frequently in Dr. Laycock's pages, that they cease to excite astonishment; "females and children," says he, "are more liable than men, indeed are alone liable, to epidemic convulsions." (p. 180.) He adds, however, we presume by way of qualification, that the mind "may be so excited by oratory, or by religious exercises, that a temporary susceptibility is developed," and he refers, as an example, to the dancing mania of the fourteenth century; but the qualification in fact would nearly swallow up the assertion, even if the latter were not, on other grounds, grossly and obviously incorrect; for it so happens that a great majority of the convulsive epidemics which have at different times occurred have been in close connexion with religious fanaticism, and, as in the instance of the dancing mania, have affected men as well as women.

With regard to *endemic* convulsions there are few cases which can properly be brought under this denomination, but these are quite unfavorable to our author's assertion. The leaping ague of some parts of Scotland may be considered as both epidemic and endemic, since it is nearly peculiar to those regions, and is chiefly prevalent at certain times. It attacks people of all ages and both sexes. Tetanus, which may in some sort be considered as an endemic convulsive disease of certain tropical countries, attacks males more frequently than females.

What then becomes of Dr. Laycock's affirmation that women and children are alone liable to epidemic or endemic convulsions? We are quite aware that he knows its inaccuracy just as well as we do, and our only wonder is what can induce him to make such random assertions.

In chap. vii. Dr. Laycock discusses the "relations of the nervous diseases of women to the nervous system in general." He commences with the recapitulation of a general law before laid down, namely, "that gradual impairment of the functions of the nervous system is preceded by exalted energy and affectibility," or, as it is more distinctly expressed when first anounced, (chap. x. part i.) "that when the functions of the motor and sensitive system are gradually impaired, there is exalted affectibility and energy observed before the paralysis." We are by no means satisfied of the universality of this law, though we admit that it applies in some instances. An objection immediately occurs to us in *amaurosis.* Is this always preceded by increased excitability of the optic nerve? So much the reverse, that Beer and others divide simple amaurosis into the case in which blindness depends on the undue exaltation of the sensibility of the optic nerve, and that in which it depends primarily on the depression of such sensibility; and every practitioner who has paid any attention to amaurotic affections, must know that in the incipient stage, some patients experience distress, and are unable to see except in shady places, while others court a glare of light. We do not think we should have to go far in quest of additional instances opposed to this law of Dr. Laycock's; fortunately, however, there is nothing practically objectionable in the application he makes of it.

"Referring," he says, "with this law in recollection to the statements in part i. respecting the influence and anatomical and physiological connexions of the ovaria, we can have no difficulty in understanding how these organs, by acting upon the dorso-lumbar portion of the cord and the respiratory ganglia, may give rise to the most varied symptoms of disease in distant and different organs, varying in intensity from the most to the least severe, and implicating the sensitive, motor, and organic nerves." (p. 184.)

The first section of this chapter treats of "the relations of sôme general paroxysmal affections to the cerebellum and contiguous ganglia," and the second, of "the anatomical relations of the cerebellum and contiguous ganglia." But this subject need not be spoken of here, as it is noticed in another article in the present Number. See the review of the "Library of Medicine."

Chap. viii. is on "the relations of nervous diseases to the extremities of the spinal cord." Dr. Laycock here insists on the fact that in hysterical or *neuremic* diseases a metastarsis of morbid action may take place from one end of the spinal cord to the other.

"For the symptoms implicating the pelvic viscera will occasionally disappear, and vomiting, dyspnœa, cough, palpitation, and cephalea supervene, to disappear in their turn, at the menstrual or hebdomadal period, with a completeness really surprising, and be replaced by menorrhagia, diarrhœa, ischuria, constipation, vesical paralysis, and neuralgic pains of the abdomen and lower extremities, or some other affection implicating organs in connexion with the dorso-lumbar portion of the cord." (p. 198.)

The accuracy of these remarks, except in as far as they relate to hebdomadal matters, will, we believe, be admitted by every observing practitioner. With respect to the portion of the cord most liable to be affected, Dr. Laycock remarks that the pelvic viscera are most frequently implicated in those women who have the reproductive system highly developed, while the thoracic viscera, or those connected with the respiratory ganglia, are most liable to disturbance in women who approach the masculine conformation, as viragoes, women past middle life, and those who have borne no children, (p. 198.) This conclusion is certainly such as one would be inclined à priori to deduce from the premises, and we think we may also say that our general observation here coincides with our author's.

Chap. ix. treats of the "relations to the lateral halves of the nervous system."

Many facts are here adduced to show that there is a difference of development in the right and left halves of the body, both with respect to the generative and other organs. In the human subject the right has evidently the predominance, and this seems to be the case with reference to some organs among mammalia, as the liver and lungs.

In all classes of animals some difference exists in the right and left organs of generation, one side preponderating in some, and the other in the other. Dr. Laycock brings forward various facts in comparative anatomy illustrative of this subject, and makes some interesting remarks, for which we regret we cannot find space.

In chap. x. our author considers the "relations of the nervous diseases of women to the pathological changes in the nervous centres." These he dismisses, judiciously we think, with a few brief remarks, and a simple allusion to the opinion of Ehrenberg, that the nervous fibres are composed of globules immediately excreted from those of the blood—an opinion which, if admitted, would remove all difficulty in comprehending how agents which alter the composition of the blood so readily affect the nervous system. Dr. Laycock conceives that the pathological changes under consideration probably consist in impaired functions of the capillaries, or molecular changes in the neurine, not amounting to organic disease, unless the operation of the exciting causes be long continued.

Chap. xi. is on "general diagnosis, prognosis, and treatment." It contains, under two sections, some judicious remarks, which being of a general character need not detain us.

Part III. We now come to the third part of Dr. Laycock's work, namely, that on "special pathology and therapeutics," and here we rejoice to be able to meet him on less debatable ground than we have hitherto been treading. The arrangement he follows is based upon special physiology, and eight chapters are respectively occupied with the nervous diseases implicating the cutaneous structures; those affecting organs in relation with the dorso-lumbar portion of the spinal cord; those affecting the liver, spleen, and pancreas; those affecting the viscera, in connexion with the respiratory ganglia; affections of the motor system in general; paroxysmal diseases; affections of the sensitive nerves; and cerebral diseases.

In the discussion of these subjects, Dr. Laycock evinces the judgment and knowledge of an accomplished physician. We think this portion of his work particularly valuable, as exhibiting a very complete and accurate delineation of the irregular forms of hysteria, so common in practice, yet so little recognized by the mass of practitioners. We trust that the perusal of Dr. Laycock's book may convince many of his brethren of what is the simple truth, that they are every day mistaking hysteria for inflammation or organic disease; frightening the patient and her friends with a dismal prognosis, when there is in fact no danger; and retarding recovery, and permanently injuring the constitution by bleeding, starving, and confinement, when fresh air, nourishing diet, and plenty of exercise would be much more to the purpose.

From the heads of chapters just enumerated, the reader will at once perceive that an attempt to analyze this portion of the work would swell the present article to more than twice the length it has already attained. It frequently happens, in fact, that it is not the best part of a book that most requires or admits of an analytical review; and we therefore trust that the author will not think we take more delight in censure than in praise, because we dismiss what we think the best part of his work with a general commendation, while we have made particular strictures on the defects of other parts.

To conclude, we think that Dr. Laycock's book, though justly open to the censure of being far too theoretical, too vague, and frequently inaccurate in its general statements, is, nevertheless, meritorious as a whole. The author gives evidence of an intimate acquaintance with his subject and of a long and thoughtful addiction to it. His style of writing is clear, and generally good; and we may add that, to his credit, he has treated in a strictly scientific and professional spirit a subject which might easily have been twisted to *popular*, in other words to self-interested ends.

